# Localized Pigmented Villonodular Synovitis of the Hip: Sudden-Onset Pain Caused by Torsion of the Tumor Pedicle

**DOI:** 10.1155/2013/862935

**Published:** 2013-11-10

**Authors:** Kiyokazu Fukui, Ayumi Kaneuji, Eriko Kinoshita, Yuhei Numata, Takayuki Nojima, Tadami Matsumoto

**Affiliations:** ^1^Department of Orthopaedic Surgery, Kanazawa Medical University, 1-1 Daigaku, Uchinada-machi, Kahokugun, Ishikawa 920-0293, Japan; ^2^Department of Pathology and Medical Laboratory, Kanazawa Medical University, Daigaku, Uchinada-machi, Kahokugun, Ishikawa 920-0293, Japan

## Abstract

Pigmented villonodular synovitis is a rare, benign, but potentially locally aggressive disease that should be considered in younger patients who present with monoarticular joint symptoms and pathology. We present the case of a 33-year-old woman with a mass arising from her right hip joint that was examined using a multimodal radiological approach. Because her clinical presentation mimicked that of synovial osteochondromatosis of the hip, surgical dislocation was performed. Histopathological examination of the resected specimen confirmed the diagnosis of localized pigmented villonodular synovitis, with the mass consisting of proliferation of fibrohistiocytic cells, abundant hemosiderin, foamy histiocytes, and occasional giant cells. Because of the presence of tumor necrosis, we hypothesize that torsion of the tumor pedicle was the cause of acute presentation.

## 1. Introduction

The term *pigmented villonodular synovitis* (PVNS) was coined by Jaffe et al. in 1941 to describe a group of localized or diffuse synovium-based lesions involving tendon sheaths, less commonly joints, and rarely bursae [1]. PVNS is a benign proliferative disorder of the synovium of unknown origin [2–10]. Localized versus diffuse forms of PVNS may cause different clinical symptoms. It typically appears as an intra-articular effusion of low intensity on both T_1_- and T_2_-weighted images because of hemosiderin deposits, with thick fibrous tissue, synovial hyperplasia, bone erosion, and preserved bone density and joint-space width [3–5]. PVNS can be demonstrated as a dark lesion on all pulse sequences of magnetic resonance imaging (MRI) because of the ferromagnetic properties of hemosiderin [11]. PVNS of the hip is a relatively uncommon disease. At any body site, PVNS has an estimated worldwide incidence of 1.8 per million cases per year; a hip is involved in 15% of those cases [12, 13]. Here we describe the case of 33-year-old woman who presented with sudden-onset hip pain and an intra-articular mass in the left hip joint. A diagnosis of PVNS should be kept in mind in younger patients who present with monoarticular arthritis, especially when it is associated with bony erosions or a soft-tissue component.

## 2. Case Report

**Figure 1:**
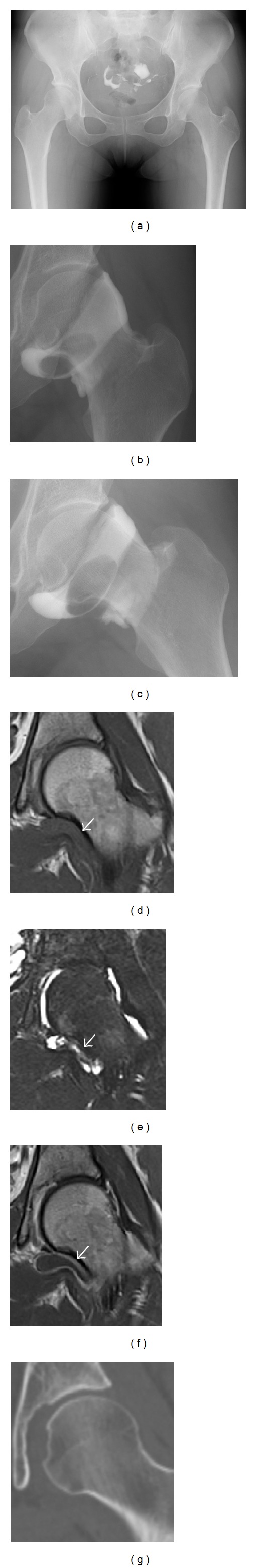
(a) An initial anterior radiograph shows no significant findings. (b, c) Enhanced stress radiographs reveal a mobile intra-articular mass. (d–f) Magnetic resonance images: (d) a coronal T_1_-weighted image shows a low-intensity mass lesion located inferior to the hip joint; (e) a coronal short *τ* inversion recovery sequence shows a mass with both low- and high-intensity areas; (f) a coronal enhanced T_1_-weighted image shows marginal enhancement of the mass. (g) Computed tomography image shows erosive changes on the fossa acetabuli and enthesis of the ligamentum teres of the femur.

**Figure 2:**
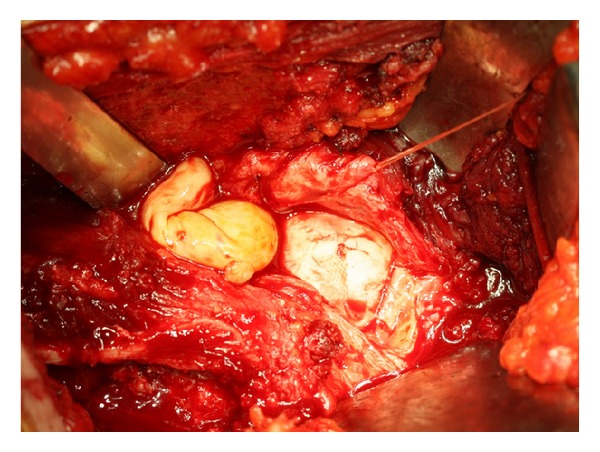
Excision of the synovial tumor using the Ganz surgical dislocation approach.

**Figure 3:**
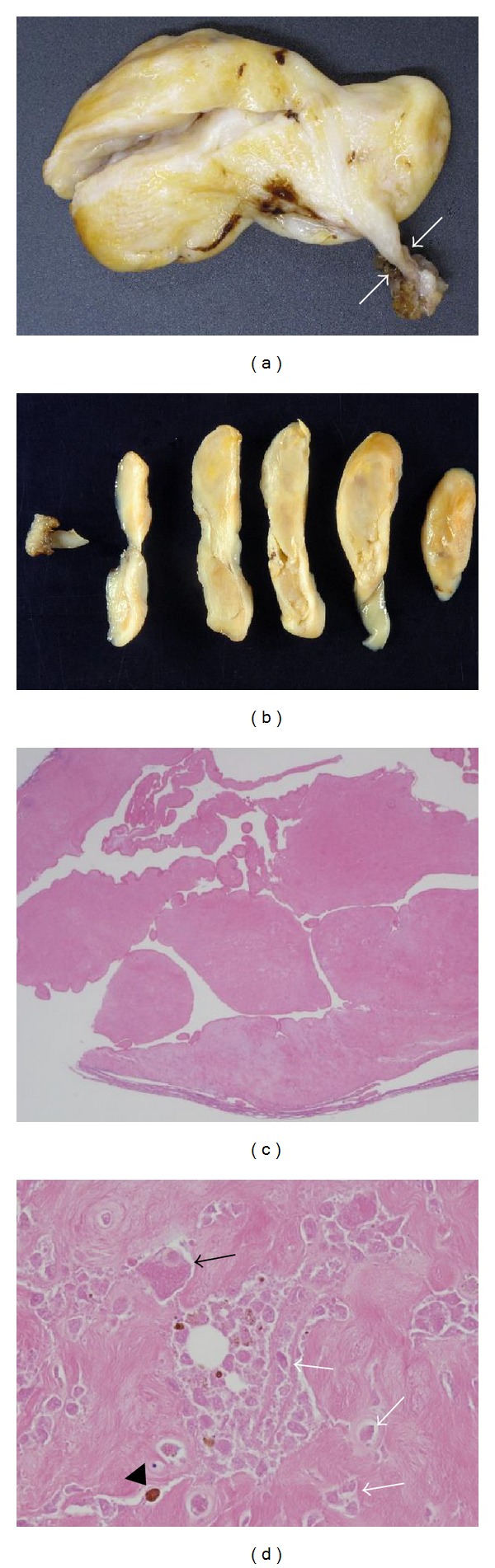
(a) The excised tumor had a pedicle (*white arrows*), and (b) the cut sections revealed tumor necrosis except in the margins. (c) A histological image shows a nodular and papillary structure and the presence of synovial cells, histiocytes, and hyperplasia (original magnification ×1.25; hematoxylin and eosin staining). (d) Histopathological findings: enucleated mononuclear stromal cells (*white arrows*) and multinuclear giant cells (*black arrow*), with hemosiderin in the cytoplasm (*black arrowhead*) (original magnification ×100; hematoxylin and eosin staining).

The patient was a 33-year-old woman who had had a mildly limited range of motion in her left hip for a long time. In October 2012, she experienced sudden, severe pain in her left hip without any antecedent trauma or episode. The pain affected her not only when she moved the hip but also when she was at rest. Her condition had been diagnosed at another hospital as synovial osteochondromatosis of the hip, for which she was given an anti-inflammatory drug (loxoprofen, 60 mg, three times daily for 10 days). Although the severe pain disappeared about 10 days after onset and only vague discomfort and pain in a specific posture had remained, she was referred to our hospital for a surgical treatment at 3 weeks after pain onset. The range of motion in the affected hip was 130° in flexion, 15° in extension, 30° in abduction, 20° in adduction, 45° in external rotation, and 15° in internal rotation. Findings on the Patrick test were positive, the anterior impingement sign was present, and she had mild tenderness of the Scarpa triangle. Preoperative blood tests revealed no evidence of diabetes, rheumatoid arthritis, infection, or abnormality in renal or liver function. Although initial radiographs, obtained 1 month after pain onset, revealed no significant findings, magnetic resonance images showed marginal enhancement of a mass located inferior to the hip joint (Figure 1). We performed surgical dislocation of the joint using the technique described by Ganz et al. [14] for tumor excision, with the patient receiving general anesthesia. We excised a whitish-yellow encapsulated tumor, 4 × 2 × 1 cm^3^, arising from the anteromedial synovium (Figure 2). We assumed that the pain was not caused by the tumor putting pressure on the surrounding area because the tumor was a soft elastic mass and could move easily. The mobility of the tumor was seen preoperatively in the enhanced stress radiographs (Figure 1). Synovectomy in the fossa acetabuli was also performed. Microscopy revealed cells of mononuclear stromal origin with hyalinization and multinucleated giant cells. There was hemosiderin pigment in macrophages and in the extracellular space. The nuclei of the mononuclear stromal cells and multinucleated giant cells had disappeared because of tumor necrosis (Figure 3).

## 3. Discussion

PVNS is a rare, benign, proliferative condition of the synovium involving a joint, bursa, or tendon sheath. Its etiology is unknown, although a neoplastic or inflammatory origin has been suggested [6, 15]. It affects patients in the age group of 20 to 40 years. Prevalence by sex has been reported to range between a 2 : 1 male-to-female ratio and a slight female preponderance [16]. Knee involvement accounts for 80% of cases, followed by the hip, ankle, shoulder, and elbow [17]. Localized PVNS is a relatively rare form, representing 27% of PVNS cases [5]. In the hip, the disease is usually of the diffuse villonodular type [18]. The average time lapse between symptom onset and diagnosis is 2 years. However, there are outliers in whom the disease was diagnosed as soon as a few days after symptom onset to as long as 12 years afterward. Localized PVNS is commonly seen in the third decade of life [3, 4], with mononuclear stromal cell hyperplasia, multinuclear giant cells, and macrophages with hemosiderin [3]. Hemosiderin can accumulate in both macrophages and extracellular compartments; its accumulation is responsible for the “pigmented” portion of the disease's name. Hemorrhage and inflammatory infiltration can take place in the tumor. The key features of PVNS seen on plain radiographs include dense soft-tissue swelling, scalloped bony erosions with sclerotic margins, and joint-space preservation during the early stage of the disease. The absence of juxta-articular osteopenia and osteophytes is characteristic [19]. Generally, sudden-onset torsion of an ovarian tumor pedicle causes severe pain in the lower abdomen [20, 21] and stagnation of the tumor's venous return, which leads to inflammation and necrosis of the tumor. In our patient, we were able to document such necrosis, and we speculate that it was responsible for the sudden onset of symptoms caused by the same mechanism of torsion as in ovarian tumor pedicles. Whether through arthroscopy or arthrotomy, excision is sufficient for localized PVNS, but if the tumor is diffuse, total synovectomy is necessary [2–5, 10]. In this case, there was no need for a biopsy to determine if it was a diffuse PVNS because we had already diagnosed it as either a localized PVNS or a synovial osteochondromatosis. Radiotherapy for PVNS is controversial [3]. We performed a Ganz surgical dislocation because it was necessary not only to excise the tumor but also to perform a synovectomy in the fossa acetabuli. In summary, sudden severe pain in the hip joint may be found, during surgery, to be caused by PVNS, a diagnosis confirmed after surgical removal of a synovial tumor. Sudden pain onset is an unusual clinical presentation for localized PVNS, so surgeons should keep in mind that it is possible that torsion of a tumor pedicle plays a role in the abruptness of symptom onset.
